# Multi-center verification of the influence of data ratio of training sets on test results of an AI system for detecting early gastric cancer based on the YOLO-v4 algorithm

**DOI:** 10.3389/fonc.2022.953090

**Published:** 2022-08-16

**Authors:** Tao Jin, Yancai Jiang, Boneng Mao, Xing Wang, Bo Lu, Ji Qian, Hutao Zhou, Tieliang Ma, Yefei Zhang, Sisi Li, Yun Shi, Zhendong Yao

**Affiliations:** ^1^ Department of Gastroenterology, The Affiliated Yixing Hospital of Jiangsu University, Yixing, China; ^2^ Microsoft Ltd Co., Suzhou, China; ^3^ Department of Gastroenterology, The Second Affiliated Hospital of Soochow University, Suzhou, China; ^4^ Department of Gastroenterology, Civil Aviation Hospital of Shanghai, A Branch of Ruijin Hospital, Shanghai, China; ^5^ Department of Internal Medicine, Yixing Maternity and Child Health Care Hospital, Yixing, China

**Keywords:** early gastric cancer (EGC), artificial intelligence (AI), convolutional neural network (CNN), ROC, YOLO, youden index

## Abstract

**Objective:**

Convolutional Neural Network(CNN) is increasingly being applied in the diagnosis of gastric cancer. However, the impact of proportion of internal data in the training set on test results has not been sufficiently studied. Here, we constructed an artificial intelligence (AI) system called EGC-YOLOV4 using the YOLO-v4 algorithm to explore the optimal ratio of training set with the power to diagnose early gastric cancer.

**Design:**

A total of 22,0918 gastroscopic images from Yixing People’s Hospital were collected. 7 training set models were established to identify 4 test sets. Respective sensitivity, specificity, Youden index, accuracy, and corresponding thresholds were tested, and ROC curves were plotted.

**Results:**

1. The EGC-YOLOV4 system completes all tests at an average reading speed of about 15 ms/sheet; 2. The AUC values in training set 1 model were 0.8325, 0.8307, 0.8706, and 0.8279, in training set 2 model were 0.8674, 0.8635, 0.9056, and 0.9249, in training set 3 model were 0.8544, 0.8881, 0.9072, and 0.9237, in training set 4 model were 0.8271, 0.9020, 0.9102, and 0.9316, in training set 5 model were 0.8249, 0.8484, 0.8796, and 0.8931, in training set 6 model were 0.8235, 0.8539, 0.9002, and 0.9051, in training set 7 model were 0.7581, 0.8082, 0.8803, and 0.8763.

**Conclusion:**

EGC-YOLOV4 can quickly and accurately identify the early gastric cancer lesions in gastroscopic images, and has good generalization.The proportion of positive and negative samples in the training set will affect the overall diagnostic performance of AI.In this study, the optimal ratio of positive samples to negative samples in the training set is 1:1~ 1:2.

## Background

Gastric cancer is the third leading cause of cancer-related mortality and one of the most common malignant tumors in the world. Over one million new cases of gastric cancer are diagnosed each year, resulting in over 780000 deaths (1, 2). The prognosis for people with gastric cancer varies according to the stage, with advanced gastric cancer patients having a poor prognosis. Patients with early gastric cancer, on the other hand, have a 5-year survival rate that exceeds 90%. This is because patients can be treated quickly following an endoscopic submucosal dissection (ESD).

In this view, endoscopy is widely regarded as the gold standard for early gastric cancer detection. However, the ability of endoscopic physicians to distinguish benign from malignant tissue *via* gastroscopy is highly dependent on their diagnostic competency, and inexperienced endoscopic physicians frequently misdiagnose patients. Atrophic gastritis is a precancerous condition that accounts for approximately 95% of stomach adenocarcinomas (3).

The morphological traits of early gastric cancer are difficult to distinguish from atrophic gastritis when using white light endoscopy. Endoscopic experts require extensive specialized training and a wide range of skills to successfully detect gastric cancer. Disparities in early gastric cancer detection rates across locations and hospital levels are related to differences in endoscopic physician expertise. Therefore, the most effective way to reduce stomach cancer mortality is to improve the effectiveness of early gastric cancer endoscopic diagnosis.

A growing number of researchers have developed an image recognition system based on a convolutional neural network for medical practice and disease screening, for example, the identification and classification of skin cancer (4, 5), radiation oncology (6), retinopathy (7), and histological classification of pathological biopsies (8).

Several studies that used CNN to train and recognize images of gastric cancer have yielded positive results (9–11). However, in this work, we used the YOLO-v4 algorithm (12)(Source code is at https://github.com/AlexeyAB/darknet.). The algorithm is a region-based CNN with high speed, adaptability, and a low rate of detection of background errors.

This multidisciplinary (AI and endoscopy) topic is attracting an increasing number of research institutes. While the field appears promising, it raises concerns about the flexibility and stability of these algorithms. When the algorithm design cannot be improved further, there is a tendency to seek new images and win through data volume.

In our previous work, we developed the EGC-YOLO with the YOLO-v3 algorithm (13) using nearly 40,000 gastroscopic images and achieved acceptable specificity and sensitivity in the test sets, but we want to go even further. In this study, we collected over 200,000 photos from four hospitals to see how proportion of positive and negative samples in the training set influenced EGC-YOLOV4 test performance.

## Methods

### Data sources and classification

We collected gastroscopic cases in Yixing People’s Hospital between 2018 and 2021 and divided them into 2 categories based on pathological types, including images of early gastric cancer and images of non-gastric cancer (including moderate + heterosexual hyperplasia, severe heterosexual hyperplasia, and intramucosal carcinoma). There were 1200 early gastric cancer images and 219,718 non-gastric cancer images. There were a total of 220918 gastroscopic images obtained. The images were selected by four endoscopists, each with more than 10 years of experience in endoscopy and more than 10000 gastroscopy cases. In addition, 568 gastroscopic images (268 early gastric cancer images and 300 non-gastric cancer images) were obtained from the Department of Gastroenterology, Nanjing Drum Tower Hospital; 1340 images (403 images of early-stage gastric cancer and 937 images of non-gastric cancer) were obtained from the Department of Gastroenterology, The Second Affiliated Hospital of Soochow University; 4453 gastroscopic images (366 for early-stage gastric cancer and 4087 for non-gastric cancer) were obtained from The Endoscopy Center of Civil Aviation Hospital of Shanghai.

### Data platform

We created an online test platform and data storage server specifically for this experiment, which has two modules. In the first module, each test worker can upload data and use the online image box selection and marking tool to mark and save each image. The platform website is https://image-exp.dev.zhishiq.com:8443/. In the second module, the results of all test sets were displayed immediately after the test. On each tested image, the sites suspected of having early gastric cancer lesions were marked with red boxes, which is very intuitive.

### Research equipment and software

**Figure 1:**
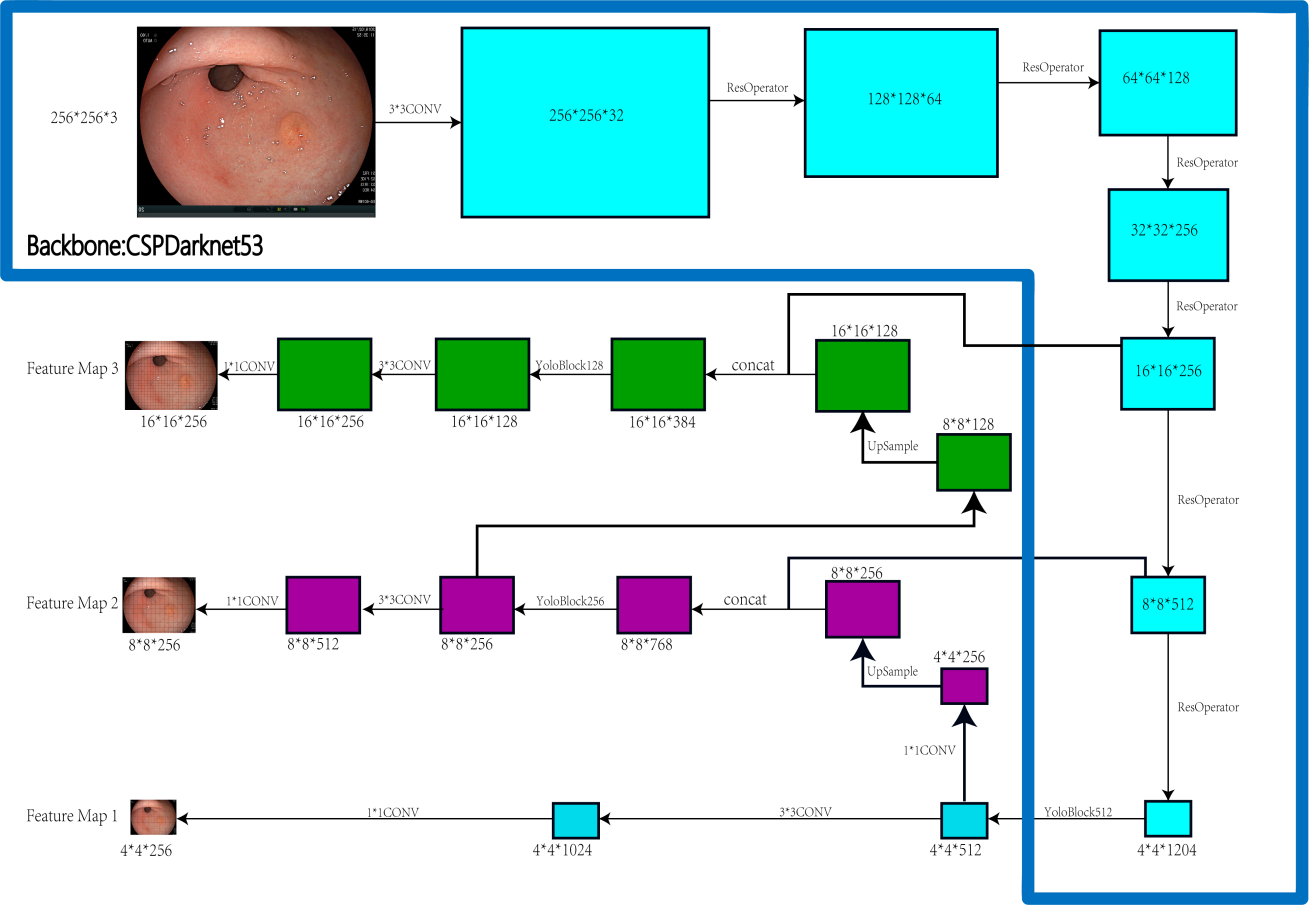
Steps and Architecture for EGC-YOLOV4.

PYTHON programming language, LINUX OS system, GPU: NVIDIA RTX 2080TI+NVIDIA GTX 1080TI. **Figure 1** depicts the construction of the EGC-YOLOV4.

### Training set construction

A total of 220918 gastroscopic images, 1200 endoscopic images of early gastric cancer, and 219718 non-gastric cancer images were provided by Yixing People’s Hospital. Hereinafter collectively referred to as training sources.

Training set 1: 1200 images of early gastric cancer were selected as positive samples from training sources, and 0 images of non-gastric cancer were randomly selected as negative samples from training sources, with a positive to negative ratio of 1:0.

Training set 2: 1200 images of early gastric cancer were selected as positive samples from training sources, and 1200 images of non-gastric cancer were randomly selected as negative samples from training sources, with a positive to negative ratio of 1:1.

Training set 3: 1200 images of early gastric cancer were selected as positive samples from training sources, and 2400 images of non-gastric cancer were randomly selected as negative samples from training sources, with a positive to negative ratio of 1:2.

Training set 4: 1200 images of early gastric cancer were selected as positive samples from training sources, and 4800 images of non-gastric cancer were randomly selected as negative samples from training sources, with a positive to negative ratio of 1:4.

Training set 5: 1200 images of early gastric cancer were selected as positive samples from training sources, and 9600 images of non-gastric cancer were randomly selected as negative samples from training sources, with a positive to negative ratio of 1:8.

Training set 6: 1200 images of early gastric cancer were selected as positive samples from training sources, and 19200 images of non-gastric cancer were randomly selected as negative samples from training sources, with a positive to negative ratio of 1:16.

Training set 7: 1200 images of early gastric cancer were selected as positive samples from training sources, and 38400 images of non-gastric cancer were randomly selected as negative samples from training sources, with a positive to negative ratio of 1:32

### Test set construction

Test set 1: This test set consists of a single external test set. All the 268 positive samples were gastroscopic images of early gastric cancer provided by the Department of Gastroenterology of Nanjing Drum Tower Hospital, whereas 300 negative samples were non-gastric cancer gastroscopic images provided by this hospital.

Test set 2: This test set consists of a single external test set. All the 366 positive samples were gastroscopic images of early gastric cancer from the Endoscopy Center of Gubei Branch of Shanghai Ruijin Hospital, whereas 4087 negative samples were randomly selected non-gastric cancer images.

Test set 3: This test set consists of a single external test set. All the 403 positive samples were gastroscopic images of early gastric cancer from the Department of Gastroenterology, The Second Affiliated Hospital of Soochow University, whereas 937 negative samples were non-gastric cancer images.

Test set 4: This test set consists of a mixed external test set. All the 717 positive samples were images randomly selected by the system after mixing images of early gastric cancer from the three hospitals mentioned above, whereas 1002 negative samples were images randomly selected by the system after mixing images of non-gastric cancer from the three hospitals.

## Results

### 1. The training set 1 model

**Figure 2:**
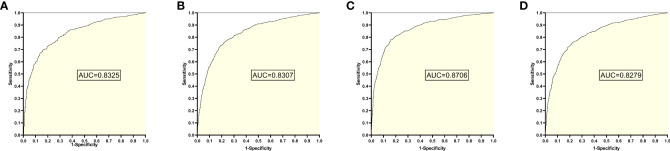
ROC curves and AUC values obtained from training set 1 model testing each test set: **(A)** results from test set 1; **(B)** results from test set 2; **(C)** results from test set 3; **(D)** results from test set 4.

The training set 1 model was tested individually on test sets 1, 2, 3, and 4, respectively, with an average reading speed of 15 milliseconds/sheet. As shown in **Figure 2**, the measured AUC values were 0.8325, 0.8307, 0.8706, and 0.8279, respectively.

### 2. The training set 2 model

**Figure 3:**
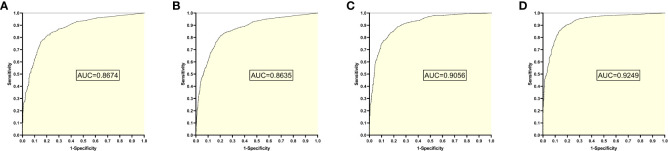
ROC curves and AUC values obtained from the training set 2 model testing each test set: **(A)** results from test set 1; **(B)** results from test set 2; **(C)** results from test set 3; **(D)** results from test set 4.

The training set 2 model was tested individually on test sets 1, 2, 3, and 4, respectively, with an average reading speed of 15 milliseconds/sheet. As shown in **Figure 3**, the measured AUC values were 0.8674, 0.8635, 0.9056, and 0.9249, respectively.

### 3. The training set 3 model

**Figure 4:**
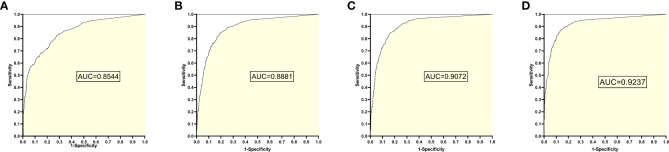
ROC Plot and AUC Values Obtained from Training Set 3 Model Testing Each Test Set: **(A)** Results from Test Set 1; **(B)** Results from Test Set 2; **(C)** Results from Test Set 3; **(D)** Results from Test Set 4.

The training set 3 model was tested separately for test sets 1, 2, 3, and 4, respectively, with an average reading speed of 14 milliseconds/sheet. As shown in **Figure 4**, the measured AUC values were 0.8544, 0.8881, 0.9072, and 0.9237, respectively.

### 4. The training set 4 model

**Figure 5:**
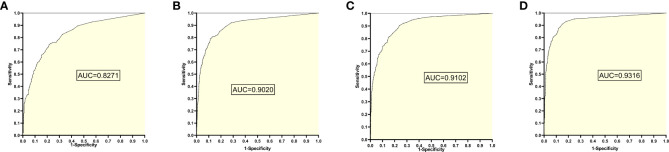
ROC curves and AUC values obtained from the training set 4 model testing each test set: **(A)** results from test set 1; **(B)** results from test set 2; **(C)** results from test set 3; **(D)** results from test set 4.

The training set 4 model was tested separately for test sets 1, 2, 3, and 4, respectively, with an average reading speed of 14 milliseconds/sheet. As shown in **Figure 5** ,the ROC curve was plotted with AUC values of 0.8271, 0.9020, 0.9102, and 0.9316, respectively.

### 5. The training set 5 model

**Figure 6:**
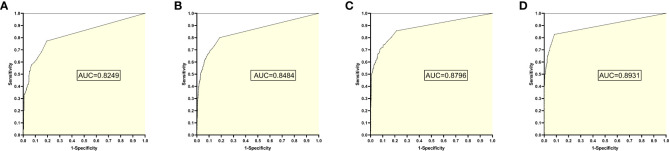
ROC curves and AUC values obtained by the training set 5 model testing each test set: **(A)** results from test set 1; **(B)** results from test set 2; **(C)** results from test set 3; **(D)** results from test set 4.

The training set 5 model was tested individually on test sets 1, 2, 3, and 4, respectively, with an average reading speed of 14 milliseconds/sheet. As shown in **Figure 6**, the AUC values were 0.8249, 0.8484, 0.8796, and 0.8931, respectively.

### 6. The training set 6 model

**Figure 7:**
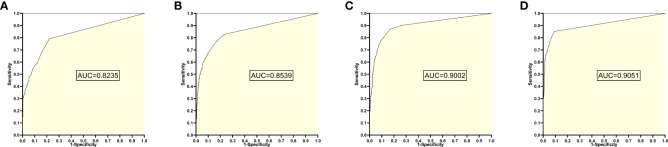
ROC curves and AUC values obtained from the training set 6 model testing each test set: **(A)** results from test set 1; **(B)** results from test set 2; **(C)** results from test set 3; **(D)** results from test set 4.

The training set 6 model was tested individually on test sets 1, 2, 3, and 4, respectively, with an average reading speed of 15 ms/sheet. As shown in **Figure 7**, the AUC values were 0.8235, 0.8539, 0.9002, and 0.9051, respectively.

### 7. The training set 7 model

**Figure 8:**
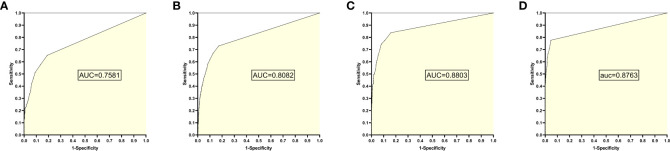
ROC curves and AUC values obtained from the training set 7 model testing each test set: **(A)** results from test set 1; **(B)** results from test set 2; **(C)** results from test set 3; **(D)** results from test set 4.

The training set 7 model was tested individually on test sets 1, 2, 3, and 4, respectively, with an average reading speed of 16 milliseconds/sheet. As shown in **Figure 8**, the AUC values were 0.7581, 0.8082, 0.8803, and 0.8763, respectively.

### 8. Change trend of AUC

**Figure 9:**
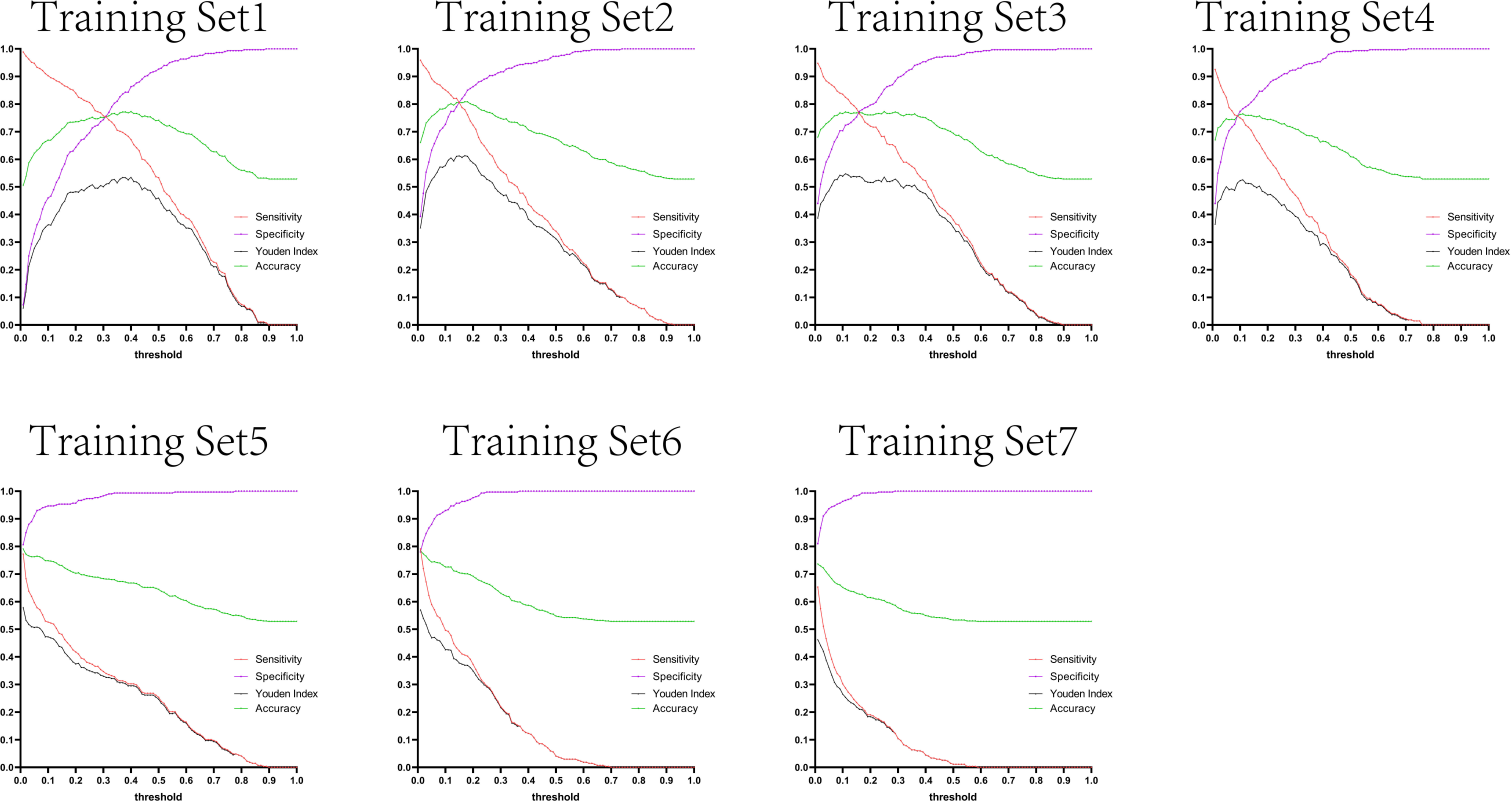
The trend lines graph of AUC value with the gradient of sample proportion in the training set models.

According to the change trend of AUC in **Figure 9**, it can be found that the AUC first increases and then decreases with the increase of negative samples in the training set model. When the training set 2 model and training set 3 model are used for the test set, the AUC results of all test sets are greater than 0.85. Even when the ratio of positive samples to negative samples in the training set is at 1:1 ~ 1:2, EGC-YOLOV4 has good diagnostic performance for each test set (with the best generalization). Although Test Set 4 is a mixture of the first three independent external test sets with complicated internal data types, after testing, it was found that its maximum AUC once exceeded 0.90, and the highest AUC of all test results could be obtained. The **Figure 8** AUC peak for test set 1 occurred in the training set 2 model, the AUC peak for test set 2 occurred in the training set 4 model, the AUC peak for test set 3 occurred in the training **Figure 8** set 4 model, and the peak for test set 4 occurred in the training set 4 model.

### 9. Trends in sensitivity and specificity

**Figure 10:**
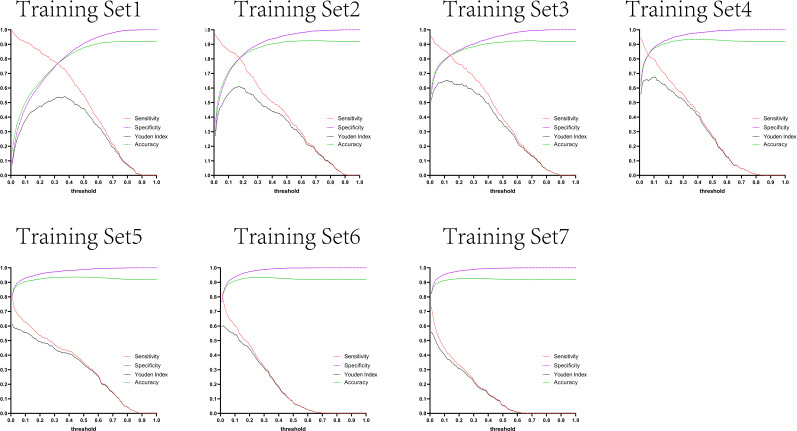
Curves of sensitivity, specificity, Youden index, and accuracy with threshold after test set 1 was tested separately by seven training set models.

**Figure 11:**
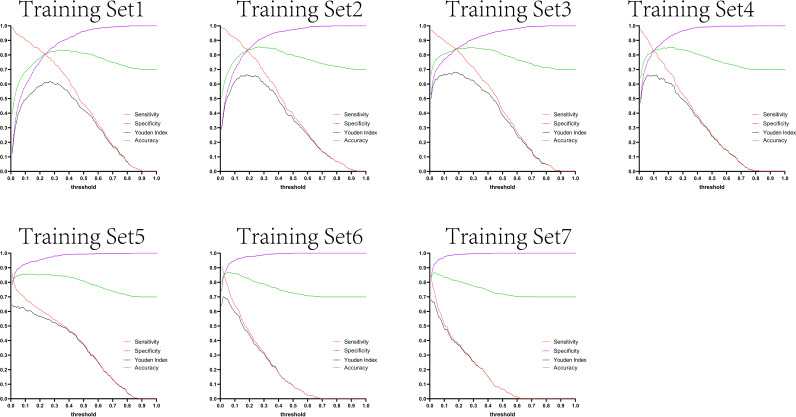
Curves of sensitivity, specificity, Youden index, and accuracy with threshold after test set 2 was tested separately by seven training set models.

**Figure 12:**
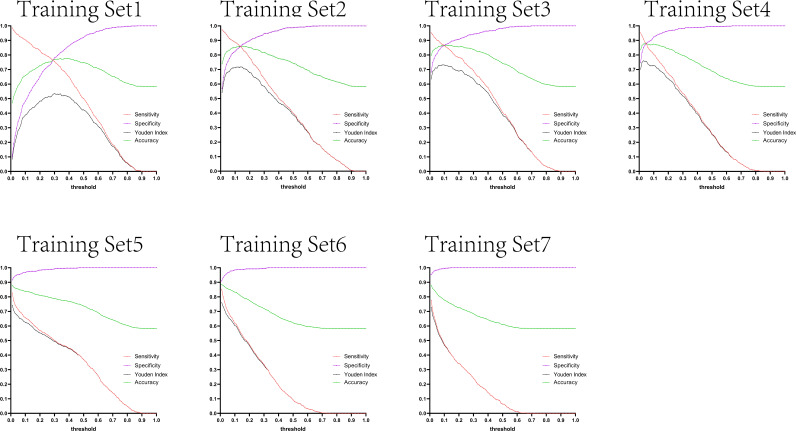
Curves of sensitivity, specificity, Youden index, and accuracy with threshold after test set 3 was tested separately by seven training set models.

**Figure 13:**
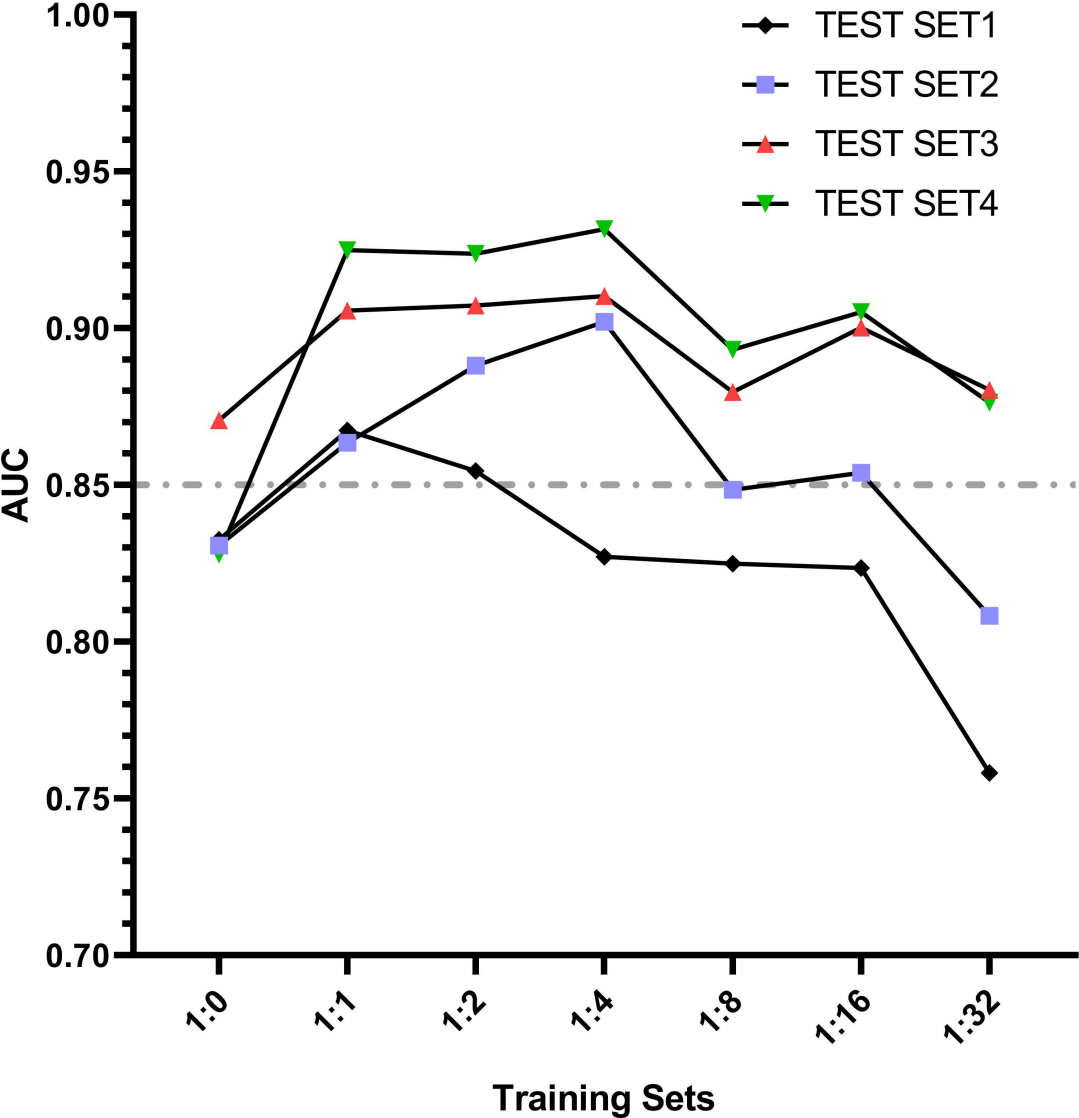
Curves of sensitivity, specificity, Youden index, and accuracy with threshold after test set 4 was tested separately by seven training set models.

The variation tendency in **Figures 10**–**13** showed that when there were few negative samples in the training set model, the minimum value of specificity changed sharply with the threshold. When there were more negative samples in the training set, the minimum value of specificity increased significantly, and its stability was significantly improved, and it could finally be very steadily maintained above 0.9. The sensitivity always showed a monotonic decreasing trend with increasing threshold, and when the negative sample in the training set model increased significantly, the maximum value of sensitivity gradually decreased and finally was lower than 0.8.

**Figures 10**–**13** are the curves of sensitivity, specificity, Youden index, and accuracy with threshold after testing test sets 1, 2, 3, and 4 with seven training set models, respectively.

### 10. Comparison of youden index and accuracy

In the test results of each group in this study, we listed the maximum Youden index and maximum accuracy rate of each group in **Tables 1** and **2**, and their corresponding sensitivity and specificity. It can be obviously found that the accuracy rate is always higher than Youden index under the same threshold value, but the corresponding sensitivity and specificity cannot be stably at a higher level at the same time when it reaches the maximum value, and some sensitivities are even less than 0.5. However, the Youden index better balances the importance of sensitivity and specificity in the actual diagnosis, that is, the corresponding sensitivity and specificity results can reach a good level at the same time when the Youden index is the largest, which ensures a low missed diagnosis rate while reducing misdiagnosis, so the Youden index is superior to the accuracy rate in the indication of threshold value.

**Table 1:** The results of testing four test sets for each of the seven training set models.

TRAINING SETS	TEST SETS	Youden index (max)	threshold	sensitivity	specificity
The training set 1 model	test set 1	0.534975	0.400	0.671642	0.863333
	test set 2	0.541106	0.370	0.729508	0.811598
	test set 3	0.620138	0.270	0.779156	0.840982
	test set 4	0.534972	0.300	0.754533	0.780439
The training set 2 model	test set 1	0.613184	0.17	0.779851	0.833333
	test set 2	0.61088	0.17	0.814208	0.796672
	test set 3	0.66403	0.18	0.841191	0.822839
	test set 4	0.718621	0.14	0.856346	0.862275
The training set 3 model	test set 1	0.547960	0.110	0.824627	0.723333
	test set 2	0.652434	0.120	0.844262	0.808172
	test set 3	0.682519	0.180	0.843672	0.838847
	test set 4	0.732363	0.090	0.873082	0.859281
The training set 4 model	test set 1	0.525870	0.110	0.742537	0.783333
	test set 2	0.686087	0.090	0.806011	0.870076
	test set 3	0.664165	0.060	0.903226	0.760939
	test set 4	0.760461	0.030	0.909344	0.851297
The training set 5 model	test set 1	0.579055	0.010000	0.772388	0.806667
	test set 2	0.613122	0.010000	0.800546	0.812576
	test set 3	0.643699	0.010000	0.856079	0.787620
	test set 4	0.743225	0.010000	0.827057	0.916168
The training set 6 model	test set 1	0.571045	0.010000	0.791045	0.780000
	test set 2	0.602643	0.020000	0.770492	0.832151
	test set 3	0.701277	0.020000	0.870968	0.830309
	test set 4	0.761344	0.010000	0.852162	0.909182
The training set 7 model	test set 1	0.462985	0.010000	0.652985	0.810000
	test set 2	0.557744	0.010000	0.729508	0.828236
	test set 3	0.679692	0.010000	0.838710	0.840982
	test set 4	0.729942	0.010000	0.776848	0.953094

Their maximal Youden index, their respective threshold values, as well as their sensitivity and specificity.

**Table 2:** The results of testing four test sets for each of the seven training set models.

TRAINING SETS	TEST SETS	Accuracy (max)	threshold	sensitivity	specificity
The training set 1 model	test set 1	0.772887	0.400	0.671642	0.863333
	test set 2	0.920728	0.740	0.161202	0.988745
	test set 3	0.833582	0.340	0.702233	0.890075
	test set 4	0.774869	0.340	0.718271	0.815369
The training set 2 model	test set 1	0.808099	0.170	0.779851	0.833333
	test set 2	0.926566	0.680	0.204918	0.991192
	test set 3	0.854478	0.260	0.751861	0.898613
	test set 4	0.860384	0.150	0.842399	0.873253
The training set 3 model	test set 1	0.772887	0.250	0.682836	0.853333
	test set 2	0.924545	0.640	0.204918	0.988989
	test set 3	0.851493	0.300	0.717122	0.909085
	test set 4	0.865620	0.100	0.864714	0.866267
The training set 4 model	test set 1	0.764085	0.110	0.742537	0.783333
	test set 2	0.935325	0.360	0.486339	0.975532
	test set 3	0.853731	0.220	0.672457	0.931697
	test set 4	0.878418	0.050	0.875872	0.880240
The training set 5 model	test set 1	0.790493	0.010000	0.772388	0.806667
	test set 2	0.936447	0.460000	0.387978	0.985564
	test set 3	0.856716	0.090000	0.702233	0.923159
	test set 4	0.878999	0.010000	0.827057	0.916168
The training set 6 model	test set 1	0.785211	0.010000	0.791045	0.780000
	test set 2	0.934651	0.300000	0.322404	0.989479
	test set 3	0.869403	0.060000	0.739454	0.925293
	test set 4	0.885398	0.010000	0.852162	0.909182
The training set 7 model	test set 1	0.735915	0.010000	0.652985	0.810000
	test set 2	0.927015	0.290000	0.248634	0.987766
	test set 3	0.867164	0.030000	0.744417	0.919957
	test set 4	0.879581	0.010000	0.776848	0.953094

Their maximal Accuracy, their respective threshold values, as well as their sensitivity and specificity.

## Discussion

In this study, we constructed and developed the EGC-YOLOV4 system with the YOLO-v4 algorithm, trained AI according to different sample ratios using gastroscopic images from Yixing People’s Hospital, obtained seven training set models, created four test sets with gastroscopic images from three different hospitals, and tested four test sets using seven training set models, respectively, which could efficiently and accurately screen for early gastric cancer in gastroscopic images. YOLO-v4 is capable of properly diagnosing gastroscopy pictures from various hospitals and has excellent generalizability. The percentage of positive and negative samples in the training set influences the overall diagnostic performance of AI, and an excessive number of negative samples decreases the diagnostic performance.

The rapid advancement of artificial intelligence’s picture identification capability has given the area of medical illness diagnostics significant technological advantages. In clinical practice, the primary diagnostic procedures for gastric cancer are gastroscopy + mucosal pathological biopsy and imaging detection, which are employed for qualitative, localisation diagnosis and staging assessment, respectively. In the last two years, AI has conducted a number of studies in the area of gastroscopic image recognition, and more and more evidence indicates that a gastroscopic AI system may be used in clinical practice.

Ishioka et al. (14), for instance, created a real-time AI system for recognizing gastric cancer picture frames in gastroscopic movies that has a sensitivity of 94.1 percent for discriminating stomach cancer. The gastric cancer diagnostic method developed by Hirasawa et al. (15) not only detects stomach cancer but also localizes it with a sensitivity of 92.2% and a positive predictive value of 30.6%. Tang et al. (16) developed a D-CNN model to predict gastric mucosal cancer using 3407 gastroscopic images from 666 gastric cancer patients as the training set and 228 gastroscopic images as the test set. The AUC of the AI model for distinguishing intramucosal cancer from advanced gastric cancer was found to be 0.942, with a sensitivity of 0.905 and a specificity of 0.853.Zhu et al. (17) constructed a CNN-CAD system based on the ResNet50 algorithm to determine the depth of gastric cancer invasion in order to screen patients undergoing endoscopic surgery. This system had an AUC of 0.94, sensitivity of 76.47 percent, specificity of 95.56 percent, overall accuracy of 89.16 percent, positive predictive value of 89.66 percent, and negative predictive value of 88.97 percent. This CNN-CAD method provides a high degree of accuracy and specificity for determining the depth of gastric cancer invasion, hence reducing the need for gastrectomy. Nagao et al. (18) used traditional white-light gastroscopy images, narrow-band imaging endoscopic images, and chromoendoscopy images to train AI to predict the depth of invasion of gastric cancer; the results demonstrated that the AI system could accurately predict the depth of invasion of gastric cancer.

Currently, the quality of endoscopic equipment and video collection devices in hospitals of all levels in China is inconsistent, high-definition acquisition cards are not widely used, and not all gastroscopic pictures can achieve 1080p resolution. Even though this field has great application potential, the stability and generalization of artificial intelligence in detecting endoscopic images from various medical institutions are still worth verifying, as overfitting issues frequently arise during the construction of AI systems, and how to obtain good generalization of AI systems is the most pressing issue at present. In order to deal with this issue, the training set we constructed in this study contains many images with varying resolutions to simulate the uneven image quality encountered in daily work. We then constructed four test sets with gastroscopic images from three different hospitals to test the diagnostic performance of each training set model separately and independently, and the results demonstrated that EGC-YOLOV4 could still demonstrate good diagnostic performance and was able to fully demonstrate its effectiveness.

People often train AI systems with more photos in the hopes of winning by volume when the algorithm architecture cannot be much further improved. But are AI systems that have been trained on more photos better tested? The findings of this investigation clearly refute this theory.The findings of this research show that an excessive number of negative samples in the training set reduces the diagnostic performance of AI, but an optimum number of negative samples may maximize AI power. The highest generalization performance in the EGC-YOLOV4 system is achieved by AI trained with a training set sample ratio from 1:1 to 1:2. To guarantee that the AI system performs the best in terms of diagnostic performance, it is thus a good idea to use pre-experiments to estimate the ratio of positive samples to negative samples in the most appropriate training set. We also discovered that when the same training set model was used for testing, the four test sets’ optimal threshold values varied from one another, and in practice, a mature AI diagnostic software had to first set the threshold when screening for early gastric cancer. The determination of the specific set values of threshold had to be preset and corrected in accordance with various source datasets before AI testing, and the appropriate training set model had to be used.

Even though our study yielded favorable test results, there are still limitations, including: 1. In this research, during the process of manually labeling the lesion site of gastric cancer, all lesions are labeled in the shape of rectangular boxes, which is likely to result in a small amount of non-gastric cancer stomach mucosa inside the rectangular box. During the process of artificial intelligence system learning, this portion of non-gastric cancer mucosal images will be misidentified as early gastric cancer by AI, and their mucosal characteristics will be extracted, leading to the possibility that the trained artificial intelligence system will misdiagnose non-gastric cancer mucosa as gastric cancer. If the labeling tool can be modified to a polygonal lasso, it will precisely match the lesion mucosal border and optimize the decrease of the original lesion boundary. 2. Since this research only employed retrospective data and photos, further multicenter prospective studies might be conducted to remove selective bias and increase the study’s reliability; 3. To analyze the trend of AI system performance when there are fewer negative samples than positive samples, we will add more sample proportion gradients in the design process of the training set model, such as 1:0.125, 1:0.25, and 1:0.5.

## Data availability statement

The raw data supporting the conclusions of this article will be made available by the authors, without undue reservation.

## Author contributions

Conceptualization, TJ, ZY and BL. Methodology, ZY. Software, BL. Formal analysis, ZY and BL. Investigation, TJ, YZ, SL, YJ, XW, YS, JQ, HZ, TM. Resources, BM. Data curation, BL. Writing—original draft preparation, ZY. Writing—review and editing, TJ, YJ. Visualization, ZY, TJ, YJ and BL. Supervision, ZY and BM. Project administration, ZY. Funding acquisition, ZY and JQ. All authors have read and agreed to the publication of this manuscript.

## Funding

This research was funded by the Foundation of Clinical Science and Technology of Wuxi, No. Q202062 and No. Q201924.

## Acknowledgments

We thank Professor Weichang Chen for his constant advice and guidance regarding the design of this project and Professor Xiaoping Zou, who provided us with valuable gastroscopic images. Additional thanks go to Bo Lu for his contribution to the construction of EGC-YOLOV4 system. Finally, I wish to express my gratitude to the three hospitals (The Second Affiliated Hospital of Soochow University, Civil Aviation Hospital of Shanghai, and Nanjing Drum Tower Hospital) for providing us with endoscopic images.

## Conflict of interest

Author BL is an employee of Microsoft Ltd, however, he was neither assigned or asked by Microsoft Ltd to participate in this research, nor did Microsoft Ltd have any interest in the current research or future application of this study. The author did not use any resource of Microsoft Ltd or work time to conduct this research.

The authors declare that the research was conducted in the absence of any commercial or financial relationships that could be construed as a potential conflict of interest.

## Publisher’s note

All claims expressed in this article are solely those of the authors and do not necessarily represent those of their affiliated organizations, or those of the publisher, the editors and the reviewers. Any product that may be evaluated in this article, or claim that may be made by its manufacturer, is not guaranteed or endorsed by the publisher.
